# Comparison of Linear Dimensions and Angular Measurements on Panoramic Images Taken with Two Machines

**DOI:** 10.5681/joddd.2009.003

**Published:** 2009-03-16

**Authors:** Tahmine Razi, Seyed Hosein Moslemzade, Sedighe Razi

**Affiliations:** ^1^Assistant Professor, Department of Oral and Maxillofacial Radiology, Faculty of Dentistry, Tabriz University of Medical Sciences,Tabriz, Iran; ^2^Assistant Professor, Department of Orthodontics, Faculty of Dentistry, Tabriz University of Medical Sciences, Tabriz, Iran; ^3^Dentist, Private Practice, Tabriz, Iran

**Keywords:** Distortion, panoramic radiography, linear dimension, magnification

## Abstract

**Background and aims:**

Panoramic radiography is a method widely used because of low absorbed dose in patients (ap-proximately 10 times less than that in the full mouth survey), reasonable cost and time. Disadvantages of this radiography technique are magnification and distortion as a result of unequal magnification, which can influence dimensional and angular measurements used in clinical dentistry to determine root length, dental arch space, relative angulations of teeth, and implant site assessment. The aim of this study was comparison of linear dimensions and angular measurements on panoramic images taken with two machines (Planmeca and Panoura).

**Materials and methods:**

Twenty radiographs taken with each apparatus from a human dry skull were scanned. Hori-zontal, vertical and angular dimensions were measured on the skull, which were compared along with the images using Corel DRAW Software, V13.

**Results:**

Independent t-test analysis showed that horizontal magnification assessed on images from Panoura was more than that from Planmeca (P < 0.00025). There were no significant differences between the two groups in vertical di-mensions (P = 0.66). Mean magnification of angular measurements assessed on images from Panoura was less than that from Planmeca (P < 0.00025). Independent t-test analysis showed that distortion of Planmeca images were more than that of Panoura. One sample t-test showed that angular measurements were more reliable than linear dimensions.

**Conclusion:**

Panoramic radiography technique can be used for evaluation of angles but it is better to use other ra-diography techniques for vertical and horizontal measurements.

## Introduction

Today dental rotational panoramic radiography technique is widely used for the purpose of dental diagnosis. ^1^ It provides information about teeth, skeletal structures, TMJ abnormalities and maxillary sinuses. ^2^ The principal advantages of panoramic images are their broad coverage of the facial bones and teeth, low patient radiation dose (approximately 10 times less than the full mouth survey), the ease of the examination and the short time required to make an image. ^3^ Dimensional and angular measurements can be used to determine the in-clination of impacted teeth, relative position of roots, restorative abutments, and implant site assessment. ^4^

One of the disadvantages of this technique is unequal magnification and geometric distortion, which cause some problems. The vertical dimension in contrast to the horizontal dimension is little altered. These distortions result from the horizontal movement of the film and x-ray source.^3,5^

As the position of an object is moved within the focal trough, the size and shape of the resultant image changes.^3^ Panoramic technique is quite sensitive to positioning errors because of relatively narrow image layer.^6^ Objects outside the focal trough are blurred, magnified or reduced in size and are sometimes distorted. The shape and location of the focal trough varies with the brand of the equipment used.^3^

The purpose of this study was to compare the linear dimensions and angular measurements of images taken with two panoramic machines: Planmeca and Panoura.

## Materials and Methods

In this descriptive study, one dry human mandible, with unknown sex, race and age, was chosen. Vertical dimension was measured from the deepest point of sigmoid notch to an anatomic projection on the inferior border of the angle of mandible by calipers (Figure 1a). For assessment of horizontal dimension at first a tangent was drawn from the posterior projection of the condyle to the angle of mandible (Line 1). Another line was drawn perpendicular to the above-mentioned line from the upper point of the lingula (Line 2). The horizontal evaluation was made by measuring the distance from the lingula to the posterior border of the ramus (Figure 1b). Angular assessment was determined by measuring the angle of the caliper arms tangential to the inferior border of mandible and posterior border of the ramus (Figure 1c) . These measurements were made for the right and left sides.


Figure 1. Vertical measurement (a); horizontal measurement (b); and angular measurement (c) made on the mandible.

**a:**
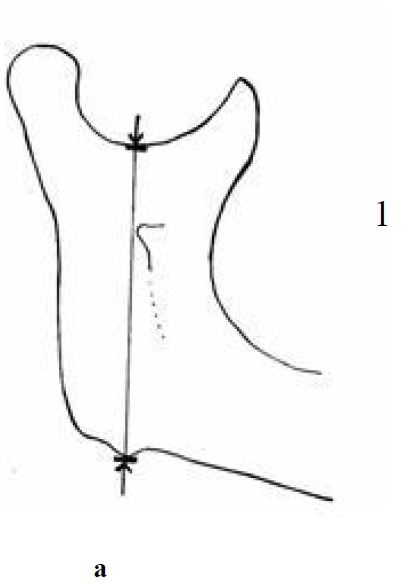

**b:**
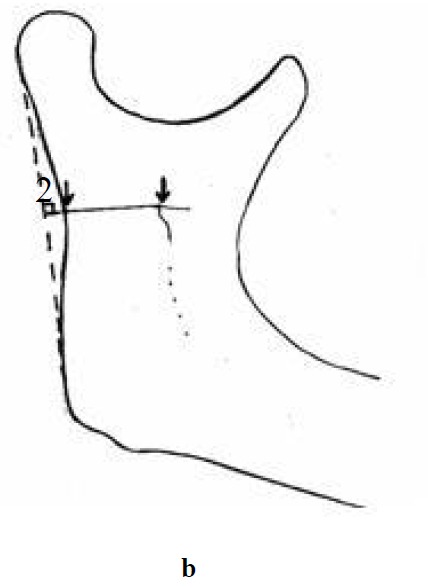

**c:**
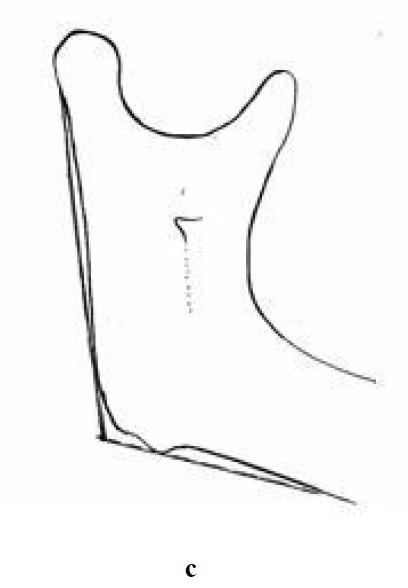

Forty exposures were provided from the selected skull. Exposure time and KVP were adjusted to the minimum conditions. Skull positioning was repeated for each image taken with either of two machines (PM 2002 CC, Planmeca, Helsinki, Finland; Proline and Panoura, Yoshida, USA) (20 images for each apparatus). Processing was carried out manually and the images were scanned.

The previously described measurements of the dry mandible were performed on scanned images using Corel DRAW Software, V13. All these evaluations were repeated by an observer after two months. The magnification of horizontal and vertical dimensions and angular measurements were analyzed using SPSS Software, V11.5.

### Statistical analysis

Independent t-test was used to compare the magnification and distortion of vertical and horizontal dimensions between the two groups. Evaluation of angular measurements was performed using one sample t-test.

## Results

The mean magnification of horizontal dimension in images taken with Panoura was more than that with Planmeca (P < 0.00025, df = 43.19, t = 3.84). The difference of the mean magnification of vertical dimension was not statistically significant (P = 0.68, df = 77, t = 0.4). The mean magnification of angular measurements in images taken with Panoura was less than that with Planmeca (P < 0.00025, df = 61.79, t = 6.65) (Table 1).

**Table 1:** Descriptive values of magnification of vertical, horizontal, and angular dimensions for each machine

Apparatus	Mean ± standard deviation
Vertical	
Planmeca	1.10 ± 0.03
Panoura	1.19 ± 0.14
Horizontal	
Planmeca	1.17 ± 0.02
Panoura	1.17 ± 0.02
Angular	
Planmeca	0.99 ± 0.01
Panoura	0.96 ± 0.01

The comparison between mean magnification of angular measurements and 1 in images taken with Planmeca showed significant difference (P < 0.0005). The comparison between mean magnification of angular measurements and 1 in images taken with Panoura showed a significant difference (P < 0.0005). The mean angular measurements calculated from images of Planmeca were closer to 1.

The comparison of the mean magnification between vertical and horizontal dimensions showed that the mean of vertical dimension in Planmeca images was more than that in horizontal dimension (P < 0.00025, df = 69.41, t = 9.87), but in Panoura images the difference was not statistically significant (P = 0.24, df = 40.63, t = −0.71).

## Discussion

The disadvantages of panoramic images are distortion and magnification, which pose problems in measurements for dental practitioners.

In the present study the mean magnification of angular measurements were more reliable than linear dimensions. The mean magnification of Planmeca images was closer to 1, which means that measurements of the skull and the images were approximately the same.

Panoramic radiography is a useful method for angular measurements.^2,7^ Larheim & Svanaes^6^ found acceptable reproducibility for the angular measurements. Wyatt et al^4^ showed no significant differences in angular measurements of various views.

Most studies have reported that patient positioning (anterior or posterior to the middle of the focal trough) influences horizontal dimension more than vertical dimension. Because of horizontal rotation of the x-ray source vertical dimension is more reliable.

In the present study, there were no significant differences in the mean vertical magnification values between the two groups. Previous studies have obtained the same results,^7,8^ but Schulze et al^9^ with digital panoramic images and Gomez-Roman et al^10^ with an orthopantomograph reported opposite findings, probably because of different machines used. As reported by Laster et al,^11^ measurements such as those assessing posterior mandibular facial symmetry may be unreliable.

In the present study, it was demonstrated that horizontal magnification of Panoura images was more than that of Planmeca. Horizontal dimensions are reported to be less reliable in comparison to vertical dimensions.,^10^ The same has been observed especially in the anterior region.,^13^

The discrepancy between the results in different studies can be attributed to the type of machine, number of rotation centers, focal trough shape, film speed, and x-ray tube head.

Comparison of vertical and horizontal magnification showed that distortion of Panoura images was less than that of Planmeca, despite claims by Planmeca manufacturer about equal magnification throughout the image. Van Elslande et al^14^ showed that magnification values reported by the manufacturer might not correspond to the calculated magnification values and might not be uniform throughout panoramic imaging area.

## Conclusion

While panoramic radiography is a valuable tool for angular assessments, other projections are considered good alternatives when greater clinical accuracy is needed for dimensional measurements.
